# Myelinated, synapsing cultures of murine spinal cord – validation as an *in vitro* model of the central nervous system

**DOI:** 10.1111/j.1460-9568.2008.06415.x

**Published:** 2008-10

**Authors:** C E Thomson, M McCulloch, A Sorenson, S C Barnett, B V Seed, I R Griffiths, M McLaughlin

**Affiliations:** 1 Comparative Anatomy and Physiology, Institute of Veterinary, Biomedical and Animal Sciences, Massey UniversityPalmerston North, New Zealand; 2 Applied Neurobiology Group, Institute of Comparative Medicine, Glasgow University Veterinary SchoolGlasgow, UK; 3 Clinical Neurosciences, University of GlasgowBeatson Labs, Glasgow, UK

**Keywords:** cytokines, MAG, multiple sclerosis, PLP–DM20, western blotting

## Abstract

Research in central nervous system (CNS) biology and pathology requires *in vitro* models, which, to recapitulate the CNS *in vivo*, must have extensive myelin and synapse formation under serum-free (defined) conditions. However, finding such a model has proven difficult. The technique described here produces dense cultures of myelinated axons, with abundant synapses and nodes of Ranvier, that are suitable for both morphological and biochemical analysis. Cellular and molecular events were easily visualised using conventional microscopy. Ultrastructurally, myelin sheaths were of the appropriate thickness relative to axonal diameter (G-ratio). Production of myelinated axons in these cultures was consistent and repeatable, as shown by statistical analysis of multiple experimental repeats. Myelinated axons were so abundant that from one litter of embryonic mice, myelin was produced in amounts sufficient for bulk biochemical analysis. This culture method was assessed for its ability to generate an *in vitro* model of the CNS that could be used for both neurobiological and neuropathological research. Myelin protein kinetics were investigated using a myelin fraction isolated from the cultures. This fraction was found to be superior, quantitatively and qualitatively, to the fraction recovered from standard cultures of dissociated oligodendrocytes, or from brain slices. The model was also used to investigate the roles of specific molecules in the pathogenesis of inflammatory CNS diseases. Using the defined conditions offered by this culture system, dose-specific, inhibitory effects of inflammatory cytokines on myelin formation were demonstrated, unequivocally. The method is technically quick, easy and reliable, and should have wide application to CNS research.

## Introduction

Research into biological and pathological mechanisms in the central nervous system (CNS) requires both *in vivo* and *in vitro* models. For *in vitro* models to be useful, they must accurately mimic the CNS *in vivo*, with respect to both the types of cells present and their abundant interactions. In the absence of such a model, many studies have to be undertaken *in vivo*, with its attendant problems.

Currently, there are several types of tissue culture models of the CNS: (i) organotypic (Bornstein, 1973; Bunge & Wood, 1973; Seil, 1979; Billings-Gagliardi *et al.*, 1980; Munoz-Garcia & Ludwin, 1986; Stanhope *et al.*, 1986; Stoppini *et al.*, 1991; Gahwiler *et al.*, 1991;Phelps *et al.*, 1996; Dusart *et al.*, 1997; Gahwiler *et al.*, 1997; Notterpek *et al.*, 1993; Park *et al.*, 2001; Ghoumari *et al.*, 2003; Birgbauer *et al.*, 2004); (ii) aggregated cell cultures (Seeds & Vatter, 1971; Matthieu *et al.*, 1978; Loughlin *et al.*, 1997; Berglund *et al.*, 2004); and (iii) dissociated primary mixed (Yavin & Yavin, 1977; Lubetzki *et al.*, 1993; Svenningsen *et al.*, 2003) or recombined (Fitzner *et al.*, 2006) cell cultures.

With respect to recapitulating the CNS *in vitro*, there are limitations to the above methods. For organotypic cultures and aggregated cultures, nutrient diffusion and gaseous exchange parameters impose cell growth limitations. Additionally, it is not easy to visualise cellular events by conventional light microscopy, due to sample thickness. Dissociated cultures are technically simple, but may yield only patchy myelination. Serum is a common additive to culture media for most methods, but as the chemical composition of serum is not completely defined, its use adds an unknown variable to the experimental regime. Because of the acknowledged difficulty in growing myelinated CNS tissue, one long-standing method of generating myelinated CNS axons has been by sourcing tissue from both the peripheral nervous system and CNS and combining them in co-culture (Wood & Bunge, 1986). This style of culture does not accurately mimic the CNS *in vitro*.

We recently described an explant culture method of murine embryonic spinal cord that yielded cultures thin enough for cellular events to be visualised by standard light microscopy and growth of myelinated fibres/axons to be monitored and measured (Thomson *et al.*, 2006). However, if axonal length is not of specific interest, then having a technique that is technically quick and easy to establish, and generates abundant myelinated axons and synapses, would be useful for both neurobiological and pathological studies.

We have developed a tissue culture model derived purely from CNS tissue, in which the cultures: (i) use media that is completely defined during myelin formation; (ii) have extensive myelin and synapse formation; (iii) are suitable for viewing with conventional light microscopy; and (iv) yield sufficient material for bulk biochemical analysis. To determine whether the technique generated a valid *in vitro* model of the CNS, two studies were performed addressing mechanistic questions in neurobiology and in neuropathology. These investigations focused on the myelin component of the cultures, as the formation of abundant myelin under defined conditions is unique to this *in vitro* CNS system. The model was used to investigate the kinetics of myelin proteins, proteolipid protein (PLP–DM20) and myelin-associated glycoprotein (MAG). The model was also used to dissect the molecular pathogenesis of inflammatory CNS diseases such as multiple sclerosis, by determining the effect on myelin of cytokines such as tumour necrosis factor-α (TNF-α), interleukin-1β (IL-1β), interleukin-6 (IL-6) and interferon-γ (IFN-γ) (Woodroofe & Cuzner, 1993; Cannella & Raine, 1995; Navikas & Link, 1996; Lock *et al.*, 2002).

This simple, robust method of culturing myelinated, synapsing CNS tissue permits easy morphological examination and generates material in quantities sufficient for biochemical studies. With abundant neurons, synapses, myelinated axons and nodes of Ranvier, this culture method should facilitate developmental, structural and functional studies in a wide variety of neuroscience areas.

## Materials and method

A more detailed protocol is available on request and will contain any updates and improvements to the method. For example, increasing the poly(l-lysine) (PLL) concentration seems to increase cell attachment.

### Tissue source

Mice were bred and killed according to UK Home Office standards. Wild-type C3H101 female mice were time-mated and embryos were collected on embryonic day (E) 13.5. The day of plugging was denoted as E0.5; thus, if a mouse was plugged overnight on Monday night (plug noted Tuesday morning), then the fetuses were used on the Monday 13 days later. Fetuses at E12.5 and E14.5 were also trialled as tissue sources. Body weight was used to monitor the pregnancy status of the time-mated female mice. The time-mated females were weighed on the day of plugging, and then every day from E10 onwards. Between E10 and E13.5, the weight of a pregnant female mouse with more than three fetuses would increase by 0.4–1.0 g/day. The average litter size at parturition of C3H101 mice was seven (average of 100 litters). For dissociated oligodendroglial cultures, neonatal mice were used on postnatal day (P) 5, where P1 was the day on which the litter was first observed.

### Tissue culture ingredients

The concentrations of nutrients noted are the final concentrations in that solution. Cell culture media, such as Hank’s balanced salt solution (HBSS) and Dulbecco’s modified Eagle’s medium (DMEM), Leibovitz’s L15 medium, and horse serum, were obtained from Gibco Invitrogen (Paisley, UK). Pig skin gelatin, Triton X-100, penicillin–streptomycin, glucose, apo-transferrin, insulin, putrescine, sodium selenite, progesterone, biotin, hydrocortisone, soybean trypsin inhibitor, DNase and bovine serum albumin (BSA) fraction V were obtained from Sigma (Poole, UK). Bovine albumin-Path-O-Cyte 4 was obtained from MP Biomedicals (OH, USA). Unless otherwise specified, all DMEM contained 1000 mg/L glucose, 100 U/mL penicillin and 100 μg/mL streptomycin.

#### Tissue culture media and reagents

##### Plating medium (PM)

The medium used to encourage attachment of spinal cord explants (Thomson *et al.*, 2006) was used for plating the dissociated spinal cord cells. This PM consisted of 50% DMEM, 25% horse serum, 25% HBSS (with calcium and magnesium), and 2 mm glutamine.

##### Differentiation medium (DfM)

This formulation was the same as the medium used to grow myelinating axons from spinal cord explants (Thomson *et al.*, 2006), and consisted of DMEM (4500 mg/L glucose), 0.5% hormone mix (see below), 10 ng/mL biotin and 50 nm hydrocortisone. In some experiments, DMEM-Advanced (Gibco) was also trialled. Insulin (10 μg/mL) was added to the DfM for the first 12–14 days of culture only, or for the full duration of culture.

##### Hormone mix

This formulation is based on the N2 mix (Bottenstein & Sato, 1979), and consisted of 1 mg/mL apo-transferrin, 20 mm putrescine, 4 μm progesterone, and 6 μm selenium.

##### PLL

PLL (molecular weight 70 000–150 000; 13.3 μg/mL) (Sigma) was used to coat the coverslips (VWR International, Lutterworth, UK) or 35-mm sterile Petri dishes (Nunclon; VWR International) as previously described (Thomson *et al.*, 2006). Three coverslips were placed in each 35-mm Petri dish for morphological studies. For biochemical studies, cells were plated directly onto the base of a PLL-coated, 35-mm Petri dish.

##### Bottenstein–Sato medium (Sato medium)

This medium was used for primary oligodendrocyte cultures. It consisted of DMEM to which was added Sato mix, giving a final concentration of 2 mm glutamine, 100 μg/mL apo-transferrin, 0.0286% bovine albumin-Path-O-Cyte 4, 0.2 μm progesterone, 100 μm putrescine, 0.45 μm thyroxine, 00.224 μm sodium selenite, and 0.5 μm tri-iodothyronine.

##### Co-culture medium

This medium was based on Sato medium but had a higher level of glucose in the DMEM (4500 mg/L) and also contained 50 nm hydrocortisone.

##### SD solution

Soybean trypsin inhibitor solution was used to stop enzymatic activity during tissue dissociation. It consisted of 0.52 mg/mL soybean trypsin inhibitor, 0.04 mg/mL bovine pancreas DNase and 3.0 mg/mL BSA fraction V made up in Leibovitz’s L15 medium.

### Culture methodology

All culture preparation and maintenance was performed in an aseptic fashion using laminar flow hoods, except for the tissue dissection stage, which was performed on the bench. Cultures were maintained in a 37°C, humidified incubator under 5% CO_2_. Myelinating cultures were fed three times weekly by replacing one-half of the medium; oligodendrocytes were fed twice-weekly replacing three-quarters of the medium each time.

#### Myelinating mouse spinal cord culture

**Fig. 1:**
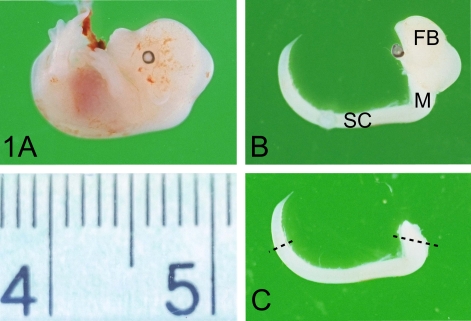
Dissection of the neuraxis from embryonic day 13.5 time-mated mouse embryos. (A) Intact embryo removed from uterus. (B) Dissected neuraxis left intact for orientation. FB, forebrain; M, myelencephalon; SC, spinal cord. (C) Isolated myelencephalon–spinal cord as dissected during culture preparation. The dotted lines indicate the portion of myelencephalon and spinal cord used in the cultures. Ruler gradations are in millimetres.

Myelinating spinal cord cultures were prepared by enzymatic and mechanical dissociation of tissue from fetuses of time-mated mice. At the appropriate stage of gestation, the pregnant mouse was killed with an overdose of halothane and CO_2_, followed by cervical dislocation. The gravid uterus was removed in an aseptic fashion into a sterile plastic Petri dish, the fetuses were extruded and decapitated, and the umbilical cord was cut. The point of decapitation was sited just caudal to the attachment of the cerebellum, about 3 mm rostral to the cervical flexure. The inclusion of the caudal part of the myelencephalon with the spinal cord in the cultures increased the amount of tissue from which cells could be harvested. Thus, the myelencephalon extending 2–3 mm rostral to the cervical flexure, and approximately 8–10 mm of spinal cord caudal to the cervical flexure, were removed from the fetus into sterile DMEM (Fig. 1). The meninges were stripped as described previously (Thomson *et al.*, 2006), and the spinal cords were transferred to the dry lid of the Petri dish, minced with a scalpel blade, and then collected into a bijou containing 1 mL of HBSS (calcium/magnesium-free) for up to six spinal cords. To dissociate more than six spinal cords, the dissociation volumes were scaled up proportionately, e.g. nine spinal cords into 1.5 mL of medium. One millilitre of 0.25% trypsin and 100 μL of 1.0% collagenase were added per mL of HBSS, and the mixture was incubated at 37°C for 20 min. One millilitre of SD solution was added per mL of HBSS, and the tissue fragments were broken down by gentle trituration (four times through a 21-gauge needle, and twice through a 23-gauge needle). The cell suspension was placed in a conical centrifuge tube, to which 5 mL of PM was added, and the cells were pelleted by centrifugation at 90 ***g***. The supernatant was discarded, and the cells were resuspended in fresh PM at 0.4 mL per cord. Ten microlitres of the cell suspension was added to 10 μL of 0.4% Trypan blue solution, and the number of live cells was counted on a haemocytometer. Additional PM was added to achieve a live cell count of 1500/μL. For morphological studies, 100 μL of the cell suspension (a total of 150 000 cells) was plated on each 13-mm PLL-coated coverslip in 35-mm Petri dishes (up to three coverslips per dish) and placed in the tissue culture incubator for 2–3 h to allow the cells to attach. For biochemical studies, 750 000 cells (500 μL) were seeded directly into the centre of each PLL-coated 35-mm Petri dish. After the cells had attached, they were fed. For coverslip cultures, the volume of PM was made up to a total of 500 μL in the Petri dish, to which 500 μL of DfM + insulin was added, ensuring that the coverslips were firmly pressed down onto the base of the dish and fully submerged in the medium. The total culture volume per dish was 1.0 mL. For cells seeded onto Petri dishes for biochemical studies, a further 250 μL of PM was added, making a total volume of PM of 750 μL per Petri dish. To this was added 750 μL of DfM + insulin (total culture volume per dish of 1.5 mL). The cultures were fed three times per week by removing approximately half of the medium and adding sufficient fresh medium to bring the total volume up to 1.0 mL per Petri dish for coverslips and 1.5 mL per dish for biochemical studies. The feeding medium was DfM + insulin for the first 12–13 days, and then DfM without insulin for the remaining time, as prolonged exposure to insulin had a negative effect (see Results). The serum used in establishing the cultures declined to negligible levels by day 14, and cultures were subsequently maintained in defined medium. Cultures were harvested at 4.5–5 weeks *in vitro* for biochemical studies, and at various times for morphological studies.

#### Mouse forebrain cultures

Embryonic forebrains were also investigated as a potential tissue source for myelinating cultures. They were harvested by splitting the calvarium dorsally with forceps and scooping out the brain using a fine spatula. The cerebral hemispheres were separated from the thalamus and the meninges were stripped. In E13.5 forebrains, the dorsal halves of the hemispheres were relatively less developed and so it was difficult to strip the meninges from them; thus, only the ventral half of the hemisphere was used. In E16.5 forebrains, the meninges were readily removed and the whole hemisphere could be dissociated. Dissociation and plating protocols were as for the spinal cords, but with scaling up of the media and enzyme volumes as appropriate.

#### Mouse oligodendrocyte cultures

Dissociated cultures of murine oligodendrocytes were prepared as described previously (Thomson *et al.*, 1999). Briefly, P5 mice were killed by halothane overdose and decapitation. The spinal cords were dissected and placed in DMEM, and the meninges were stripped. The cords were transferred to the lid of a sterile Petri dish and minced with a scalpel blade. The minced tissue was transferred to a bijou containing HBSS without calcium or magnesium (three cords/mL). To this was added 0.25% trypsin (1 mL/mL HBSS) and 1.0% collagenase (100 μL/mL HBSS). The suspension was incubated at 37°C for 20 min, at which time a second pulse of trypsin and collagenase was added. After a further 20 min of incubation, enzymatic activity was terminated by adding 1 mL SD/mL HBSS, the suspension was triturated using a syringe and needle, and the cells collected by centrifugation as for the embryonic spinal cords. The cells were resuspended in DMEM/10% fetal bovine serum (1 mL per cord) and plated onto PLL-coated 13-mm glass coverslips (100 μL per coverslip) in 24-well dishes or 35-mm plastic Petri dishes (500 μL per dish). The cells were allowed to attach for 2 h in the incubator, and were fed with 0.5 mL per well or 1.5 mL per dish of Sato medium. Cells were harvested at 7 days *in vitro* for morphological or biochemical studies.

#### Rat cultures

The above protocol for producing myelinated CNS cultures was also trialled in rats, except that rat fetuses were used at E15.0–15.5, which represents an equivalent stage of development to mouse E13.5 (http://embryology.med.unsw.edu.au/OtherEmb/Mouse.htm).

Spinal cord cells were dissociated and plated onto the coverslips in DfM as for mouse cultures.

#### Cytokine treatment of cultures

The effects of cytokines, TNF-α, IL-6, IFN-γ and IL-1β, on myelination were studied. Cytokines were obtained from R&D Systems, resuspended in DMEM with 0.1% BSA, and used at final concentrations of 5 and 20 ng/mL for TNF-α, IL-6, and IL-1β. IFN-γ was added at 250 and 1000 U/mL. Cultures were treated with cytokines from day 15 to day 25 *in vitro*.

At the end of treatment, cultures were fixed and immunostained for myelin proteins PLP–DM20 and axons (phosphorylated neurofilament), and degree of myelination was evaluated as described below.

### Morphological analysis

#### Immunostaining

The following antibodies (with their dilutions) were used: O10 against extracellular epitope of proteolipid proteins PLP–DM20 (1 : 5) (Jung *et al.*, 1996); anti-myelin basic protein (MBP) (1 : 500; N. P. Groome, Oxford, UK); AA3 against cytoplasmic and intramembranous PLP–DM20 (1 : 10) (Yamamura *et al.*, 1991) from S. Pfeiffer, University of Connecticut, USA); SMI 31 against phosphorylated neurofilament (1 : 1500; Affiniti, Exeter, UK); neurofascin (1 : 1000; gift from P. Brophy, UK); anti-caspr 1 (1 : 1000; gift from E. Peles, Israel); anti-β-tubulin III (1 : 200; Sigma). Secondary antibodies were obtained from Southern Biotech (Cambridge Bioscience, Cambridge, UK), and included goat anti-(mouse IgM) (1 : 50); goat anti-(rat IgG)–fluorescein isothiocyanate (1 : 50); goat anti-(mouse IgG_1_)–Texas-red (1 : 80) and goat anti-(mouse IgG_2b_) (1 : 40). Goat anti-(mouse IgG)–Cascade Blue (1 : 80) was obtained from Molecular Probes (Leiden, the Netherlands). All procedures were performed at room temperature unless otherwise stated.

Immunostaining and electron microscopy (EM) techniques were as previously described (Thomson *et al.*, 2006). For surface labelling with O10 antibody, live cells were rinsed briefly in DMEM and the primary antibody, diluted in DMEM, was applied for 30 min. The coverslips were washed in DMEM for 1 min, and the secondary antibody, diluted in DMEM, was applied for 30 min; coverslips were washed in DMEM for 1 min, washed briefly in phosphate-buffered saline (PBS), and then fixed in 4% paraformaldehyde/PBS for 15 min. After fixation, to label cytoplasmic antigens, cultures were washed in PBS three times for 20 min each, permeabilised with 0.5% Triton X-100 in PBS for 30 min, and blocked using a blocking buffer (0.2% pig skin gelatin, 0.1% Triton X-100 in PBS) for 30–60 min; primary antibody, diluted in blocking buffer, was then added and left on overnight at 4°C. On the following day, the coverslips were washed in PBS three times for 20 min each; the secondary antibody diluted in blocking buffer was added for 1–2 h. The coverslips were washed in PBS for 5 min, washed briefly in distilled water, mounted in AF1 glycerol/PBS (Citifluor, London, UK), and sealed using clear nail varnish.

#### Plastic sections and ultrastructure

To obtain sections for EM, cultures were fixed in strong fixative solution (2% paraformaldehyde/5% glutaraldehyde in 0.08 m sodium cacodylate buffer, pH 7.2), and the myelinated axons were harvested as described previously (Thomson *et al.*, 2006). Briefly, a 3% solution of agar (type VII, low gelling temperature; Sigma) was made up at 65°C, and a drop was placed on the lid of a clean Petri dish and allowed to gel. The culture material was removed as an intact sheet from the coverslip by gentle scraping and flushing with PBS while being viewed under a dissecting microscope. The culture material was balled up and placed on the drop of set agar, and a second drop of liquid agar was placed on top and allowed to set. The agar–culture sandwich was trimmed and left in strong fixative solution until it could be processed routinely for EM.

#### Light microscopy and photography

Light microscopy of the cultures was performed on an Olympus IX70 research fluorescence microscope with standard epifluorescence optics. For EM, thin sections (70 nm) were evaluated on a JEOL JEM-100CX II electron microscope. Images for analysis were digitally captured.

#### Quantification

##### Evaluation of myelination

**Fig. 3:**
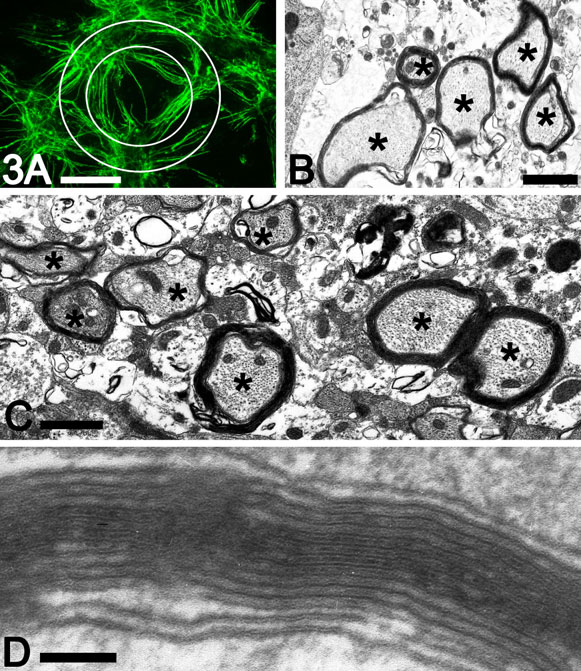
Myelin sheath formation in culture. (A) To indicate how much myelin forms in different experiments, a grid was superimposed on images and the number of myelinated axons crossing each grid line was counted. This gives an indication of myelin sheath formation, not absolute numbers of sheaths formed. For comparison between experiments, the same grid and computer settings must be used. Using the depicted grid, the average number of myelin sheaths per image was 42.3 ± 10.24 (average plus standard deviation, *n* = 216 images). (B and C) Electron micrographs depicting multiple myelinated axons indicated by asterisks (*) in transverse section at 28 days *in vitro*. (D) Electron micrograph depicting the periodicity of the cultured myelin sheaths. Scale bars: A, 200 μm; B and C, 1 μm; D, 100 nm.

Digital images were captured of non-overlapping, consecutive microscope fields of the myelinated cultures. The high density of myelinated axons made it too difficult to count the number/image. Consequently, a grid was superimposed on the images and the number of myelinated axons (as represented by myelin sheaths) crossing the grid lines was counted, summed and averaged. The number of sheaths counted depended on the grid pattern used and the resolution of the computer. To establish a baseline of the number of myelinated axons produced by the culture method, 20–25 images per coverslip, three coverslips for each sample, from 13 separate experiments were counted (total 20–25 images × three coverslips × 13 experiments). For experiments in which the effects of cytokines on myelination were examined, three coverslips per condition were used and three experimental repeats were performed. The data were expressed as percentage myelinated axons counted in cytokine-treated samples relative to the non-treated control. To obtain the baseline and cytokine trial data, the same grid and computer were used, and all counts were performed by the same operator. For the data associated with Fig. 3A, a different computer and grid were used, counted by the same operator.

##### G-ratios

Thin sections (70 nm) and diffraction gratings were photographed at a magnification of 8000. G-ratios (axonal diameter to total myelinated fibre diameter) of 83 myelinated fibres, from three different cultures at 33 days *in vitro*, were calculated using Image-Pro Plus 4.0 software (Media Cybernetics, Silver Springs, MD, USA).

### Statistical analysis

Underpinning the analysis of these results is the observation that the trials were independent of each other and shared no common elements.

Counts of myelinated axons crossing lines of a superimposed grid were performed to assess the number of myelin sheaths produced under various culture conditions. All counts were performed by one operator. To establish the reliability of this method for assessing the number of myelin sheaths, images from the myelinating cultures were selected at random and counted repeatedly over 1 week by the same observer. That observer was blinded as to the results of previous counts. The data were analysed, and the coefficient of variation of the mean of these readings was determined as being less than 0.040 (4%) over this series.

To determine the yield of myelinated axons produced by the culture method, the numbers of myelinated axons crossing superimposed grid lines were counted for 13 experimental repeats (20+ images per coverslip, three coverslips per experiments, 13 experiments). The data were analysed using standard statistical techniques to obtain the mean and the standard deviation of myelin sheath counts. These data formed the baseline dataset of normal myelin counts (referred to as ‘baseline-13’) against which was compared the results of the cytokine trials. The coefficient of variation of the mean myelin sheath count was also determined from these 13 experimental repeats.

Student’s *t*-test was used to compare data from control samples in the cytokine trials with the baseline-13 dataset of normal values to verify that the cytokine control sample results were consistent with the expected value established by the baseline-13 dataset. If the data were consistent, then the results from the cytokine trial replicates could be compared with each other. In all cases, the null hypothesis, stating that the mean myelin sheath count for each control sample in the cytokine trials and the mean of baseline-13 dataset myelin counts were from the same population, was accepted. This provided a solid basis for combining the results of the cytokine trials and for comparison of the results of cytokine repeat experiments with each other.

In assessing the effects of the cytokines on the development of myelin sheaths in the cultures, the mean number of myelin sheaths for each test sample was compared to the mean of the control sample using Student’s *t*-test, Again, the null hypothesis (that the mean value of each individual cytokine treatment would be no different from the mean of control samples) was tested. The null hypothesis was rejected, and an estimate of significance of the difference is provided.

The effects of different cytokines, and cytokine doses, were also compared with each other. In this case, the mean myelinated axon counts were again compared using Student’s *t*-test, to examine the null hypothesis that the effect of the cytokine (or its concentration) was the same as that of a different cytokine or different concentration. If the null hypothesis was rejected, an estimate of significance of the difference is provided.

In each case, the *t*-statistic determined was generated on the assumption of unpaired, unequal sample sizes and the assumption that the variances of the populations from which each of the samples were taken were unequal.

Calculations were performed using a Corel Quattro Pro X3 spreadsheet (Corel Corporation, Ottawa, Canada; http://www.wordperfect.com and further analysed using GraphPad Prism v. 5.00 for Windows (Graphpad Software, San Diego, CA, USA; http://www.graphpad.com).

### Biochemical analysis of myelinating co-cultures

Quantitative and qualitative analysis of myelin was performed on myelinating cultures at 33 days *in vitro*. Extracts of myelin from these cultures were compared to extracts of myelin-like membrane obtained from primary dissociated oligodendrocytes cultured for 7 days. To monitor growth in the myelinating and oligodendrocyte cultures, parallel cultures were established simultaneously on coverslips (for morphological evaluation by immunostaining) and in 35-mm Petri dishes (for biochemical evaluation). A typical primary oligodendrocyte culture was prepared from five or six pups, which yielded sufficient material for six to eight Petri dishes. An average of between five and seven embryos was obtained from each pregnant mouse, which generated a sufficient cell density for four to six Petri dishes per preparation.

#### Cell radiolabelling

Myelin proteins were radiolabelled with [^35^S]methionine/cysteine using a pulse-chase protocol that was developed for radiolabelling primary dissociated oligodendrocytes (McLaughlin *et al.*, 2006), with the following modifications. Myelinating cultures or primary oligodendrocyte cultures were incubated for 30 min in serum-free HBSS, which was then replaced with HBSS containing 100 μCi per mL of [^35^S]Promix (GE Healthcare Ltd) for 1 h. After this pulse period, the ^35^S-containing medium was removed, the dishes were rinsed with HBSS, fresh culture medium was added, and the dishes were returned to the incubator for an additional 18 h. After the chase period, the dishes were rinsed twice in chilled PBS, snap frozen in liquid nitrogen, and stored at −80°C until required for biochemical analysis.

#### Myelin extraction

A lipid-rich fraction was extracted from myelinating cultures or dissociated oligodendrocytes. A true myelin fraction can be isolated from the myelinating cultures, whereas only a myelin-like fraction can be isolated from dissociated oligodendrocyte cultures, as there are no axons in these cultures about which myelin sheaths can form. For simplicity, fractions from both types of culture will be referred to as ‘myelin fractions’.

The myelin fraction was extracted from the cultured cells using a modified protocol for extracting myelin from CNS tissue (Yool *et al.*, 2001). Briefly, 100 μL of chilled 50 mm Hepes (pH 7.4), supplemented with protease and phosphatase inhibitors, was added to each 35-mm dish, the cellular material was scraped off the dish, and the resulting cell lysate was syringed several times through a 26-gauge needle. A fraction of the lysate was retained to represent the total homogenate, and the remainder was mixed with 0.85 m sucrose solution in a total volume of 2 mL. The homogenates were transferred to a centrifuge tube, gently overlayered with 1 mL of 0.25 m sucrose, and centrifuged at 75 000 ***g*** for 1.5 h at 4°C using a Beckman SW50.4 rotor. The lipid-rich, myelin fraction was visible at the 0.85/0.25 m sucrose interface. It was harvested and transferred to a 2-mL tube. The myelin fraction was subjected to two rounds of hypotonic shock by the addition of five volumes of chilled distilled H_2_O, and the myelin extract was pelleted by centrifugation for 30 min at 13 000 ***g***. The final pellet was resuspended in 50 mm Hepes supplemented with protease and phosphatase inhibitors, and the protein concentrations of the total homogenate and myelin fraction were determined using the BCA protein assay system (Pierce Ltd, Perbio Science UK Ltd, Northampton, UK).

#### Immunoprecipitation and western blotting

Western blotting and immunoprecipitation procedures have been described in detail previously (McLaughlin *et al.*, 2002, 2006). The amount of material used in each reaction was dependent on the quantity of protein recovered in each fraction. Each immunoprecipitation reaction was conducted using a protein ratio of 20 : 1 for the total homogenate/myelin fraction, which typically equated to 50 μg and 2.5 μg respectively. Western blot analysis was conducted using 20 μg and 1 μg of each fraction when available.

## Results

### Characterisation of cultures from embryonic mouse spinal cord

After harvesting the dissociated cells by centrifugation, we found that the optimal plating density was 150 000 (up to 200 000) cells in 100 μL of medium per 13-mm coverslip. Within 2 h of plating, the cells had attached to the coverslip and were starting to extend short, fine processes. Within 24 h of plating, there was obvious process development, and within the first week, long processes had grown out from the cell bodies and were forming a dense reticulated pattern on the coverslip.

**Fig. 2:**
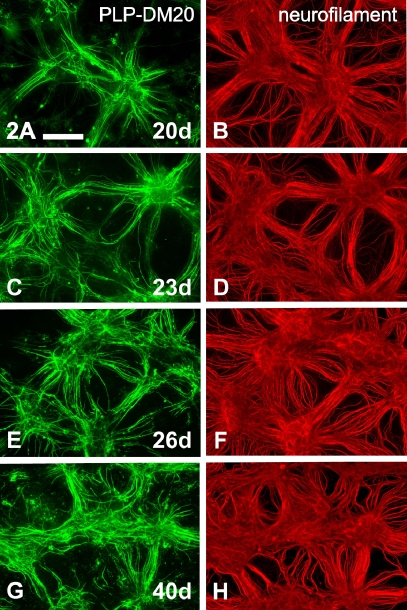
Myelination increases with time in culture. Co-labelled images stained for proteolipid protein (PLP)–DM20 (A, C, E and G) and axonal phosphorylated neurofilament (B, D, F and H) at 20 days (A and B), 23 days (C and D) and 26 (E and F) or 40 days (G and H) days *in vitro*. All time frames were harvested from the same culture. Scale bar: 200 μm.

Myelination began around day 15 *in vitro* on a few axons; however, the major wave of myelination occurred between days 17 and 23 *in vitro*, after which time the extent of myelination increased with respect to both numbers of axons and sheath thickness (Fig. 2).

The optimum time at which to study the myelinated cultures morphologically was between 4.5 and 5 weeks. At this time, the cultures were dense and axons had prominent myelination. After 5–6 weeks in culture, axons could start to deteriorate, although some cultures remained viable and healthy for 9+ weeks.

Surface staining of living, myelinating cultures with O10 antibody was attempted; however, only the top layer of myelin and cells was immunolabelled (data not shown). Although these cultures were only a few cell layers thick, the failure of antibodies to penetrate non-fixed and non-permeabilised cultures would suggest that a dense network of fibres and/or extracellular matrix develops.

**Fig. 4:**
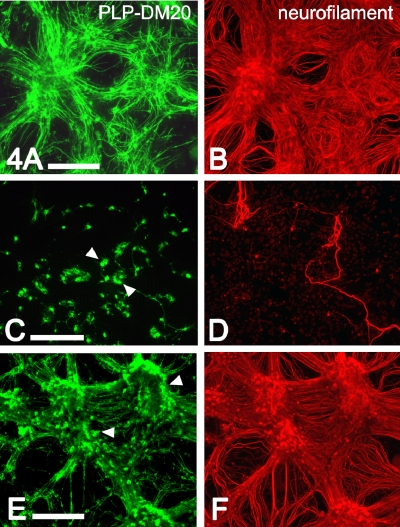
Culture integrity was determined by media composition. Myelinating cultures were grown in different media and harvested at 32 days *in vitro*, fixed and immunostained for proteolipid protein (PLP)–DM20 (A, C and E) and axonal phosphorylated neurofilament (B, D and F). (A and B) Cultures grown in standard differentiation medium. (C and D) Cultures grown in differentiation medium using DMEM-Advanced (Gibco) as a basal medium. (E and F) Cultures grown in co-culture medium. Scale bars: 200 μm. Arrowheads indicate cell bodies.

**Fig. 5:**
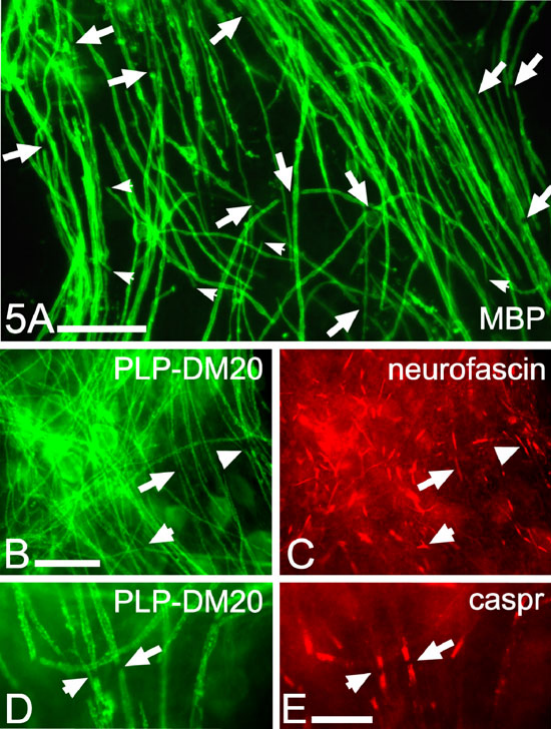
Numerous nodes of Ranvier form in myelinating cultures. Cultures were stained at 34 days *in vitro* for myelin basic protein (MBP) (A) or proteolipid protein (PLP)–DM20 (B and D) and co-stained for neurofascin (C) or caspr (E). Large arrows in A indicate nodes of Ranvier; small arrows indicate heminodes. Similar-shaped arrows indicate nodes of Ranvier and neurofascin in the perindodal area (B and C) or nodes of Ranvier and caspr in the paranodes (D and E). Scale bars: A, 25 μm; B, 20 μm; E, 10 μm.

Analysis of fixed cultures immunostained for myelin antigens such as PLP–DM20 or MBP (Figs 2, 4, 5 and 8) identified the production of large numbers of myelinated axons, extending across the whole of each culture. The extent of myelination made it difficult to count the number of sheaths per field. Consequently, we devised a quicker method to indicate the number of myelin sheaths per microscope field. This method also allowed us to evaluate the effect of factors that might promote, or inhibit, myelin sheath formation. To establish a baseline dataset, the numbers of myelinated axons crossing a superimposed grid were counted on approximately 20 images obtained from each of three coverslips for 13 different experimental repeats (therefore, the baseline-13 dataset was derived from a total of 800 images). The average number of myelinated axons crossing a set of grid lines/image in the baseline-13 dataset was 21.05 ± standard deviation 4.43, with an SEM of 1.23 and a coefficient of variation of 0.21. This is a conservative reflection of the number of myelin sheaths in culture, as sheaths that occur in a bundle/fascicle could not be counted, due to superimposition. The number of myelin sheaths counted depends on both the design of the superimposed grid and the resolution of the computer. Figure 3A was generated on a computer with a different resolution to the one on which the baseline-13 dataset and the cytokine trials were analysed. Using the grid depicted in Fig. 3A, the number of myelinated axons crossing the two concentric circles was re-counted for six separate experiments. Counts were performed on a total of 12 contiguous, non-overlapping images from each of three coverslips (a total of 36 images per experiment, six experimental repeats). The number of sheaths crossing the grid/image as shown in Fig. 3A was 42.3 ± 10.24 (average plus standard deviation).

Ultrastructural evaluation of the myelin was performed on myelinating cultures that had been harvested at 4–5 weeks *in vitro*. Myelin, with well-developed periodicity, was readily observed surrounding the axons (Fig. 3B–D). The average G-ratio for the cultured myelinated fibres was 0.76 ± 0.071 (average ± standard deviation).

Wild-type C3H101 mice were used throughout this study. Other mouse strains were also used in preliminary investigations. Successful cultures were obtained from the naturally occurring *Mbp* mutant, *Shiverer*, and from the transgenic *Plp*-null mouse. In the *Shiverer* cultures, many axons were lightly ensheathed, as indicated by positive immunostaining for PLP–DM20 with appropriate negative immunostaining for MBP (data not shown).

Embryonic age was found to be a critical factor in obtaining myelinating cultures. Spinal cord was harvested from E12.5, E13.5 and E14.5 embryos. The outcomes from each age were markedly different, with E13.5 being adopted as the standard age of tissue source. The average number of cells derived from one E13.5 spinal cord was 1 200 000, which was sufficient for approximately 8 × 13-mm coverslips, each plated with 150 000 cells. These data were derived from 106 cords harvested over 15 consecutive experiments. Comparatively, the number of cells derived from E12.5 was approximately 50% more, but myelination of axons during subsequent culture was delayed by 4–5 days. Conversely, in cultures established from E14.5 cords, the live cell yield after dissociation was markedly reduced (approximately 50% less).

As for explant cultures (Thomson *et al.*, 2006), cell survival and myelination were markedly affected by the composition of the medium. Hydrocortisone (Warringa *et al.*, 1987; Kumar *et al.*, 1989; Chan *et al.*, 1998) was added to all media as described previously (Thomson *et al.*, 2006). Similar to the situation observed in explant culture (Table 1) (Thomson *et al.*, 2006), insulin was found to be helpful if present in the medium during the first half of the culture period, but if left in for the full duration of the culture, promoted excessive numbers of oligodendroglia. These additional cells tended to obscure the myelin sheaths. DMEM-Advanced was also trialled as a base medium for the differentiation medium, but survival of both neurones and oligodendrocytes was poor in this medium (compare Fig. 4A and B with Fig. 4C and D). Co-culture medium resulted in reasonable neuronal and axonal growth, but thin myelin sheaths with intense staining of oligodendrocyte cell bodies (Fig. 4E and F). These observations suggest reduced translocation of the myelin protein isoforms, PLP–DM20, to the sheath. Previously, we have tested neurobasal medium with B27 supplement on spinal cord explants, but it did not support obvious myelin formation (Thomson *et al.*, 2006), and so was not tested in the dissociated cultures.

**Table 1:** Comparison between mean values of myelin sheath counts after adding different cytokines or cytokine doses, utilising combined results from three experimental repeats

	IL-6, 5 ng/mL (*n* = 163)	IL-6, 20 ng/mL (*n* = 172)	TNF-α, 5 ng/mL (*n* = 179)	TNF-α, 20 ng/mL (*n* = 177)	IL-1 β, 5 ng/mL (*n* = 180)	IL-1 β, 20 ng/mL (*n* = 180)	IFN-γ, 250 U/mL (*n* = 178)
IL-6, 20 ng/mL (*n* = 172)							
*t*-Value	0.267						
*P*-Value	0.789^NS^						
TNF-α 5 ng/mL (*n* = 179)							
*t*-Values	0.237	0.533					
*P*-Values	0.813^NS^	0.594^NS^					
TNF-α 20 ng/mL (*n* = 177)							
*t*-Values	12.295	11.735	14.657				
*P*-Values	< 0.0001**	< 0.0001**	< 0.0001**				
IL-1 β 5 ng/mL (*n* = 180)							
*t*-Values	1.429	1.138	1.858	11.391			
*P*-Values	0.154^NS^	0.256^NS^	0.064*	< 0.0001**			
IL-1 β 20 ng/mL (*n* = 180)							
*t*-Values	4.956	4.606	5.916	8.299	3.655		
*P*-Values	< 0.0001**	< 0.0001**	< 0.0001**	<0.0001**	< 0.0003**		
IFN-γ 250 U/mL (*n* = 178)							
*t*-Values	11.222	11.686	13.390	1.606	10.235	6.981	
*P*-Values	< 0.0001**	< 0.0001**	< 0.0001**	0.109^NS^	< 0.0001**	< 0.0001**	
IFN-γ 1000 ng/mL (*n* = 176)							
*t*-Values	18.594	17.704	22.692	8.986	18.092	16.015	16.015
*P*-Values	< 0.0001**	< 0.0001**	< 0.0001**	< 0.0001**	< 0.0001**	< 0.0001**	< 0.0001**

IL-6, interleukin-6; TNF-α, tumour necrosis factor-α; IL-1β, interleukin-1β; IFN-γ, interferon-γ; ^NS^not significant; *not quite significant; **extremely significant.

Nodes of Ranvier and sequential internodes developed along the axons in these cultures. Using myelin-specific stains (PLP–DM20 or MBP), the nodes could be seen as small breaks in the myelin sheath (Fig. 5A, B and D, large arrows); heminodes were also detected (small arrows). The molecules expressed in the nodal region of these cultured fibres mimicked those observed *in vivo*. Specifically, both caspr and neurofascin were clustered around the nodes (Fig. 5C and E).

**Fig. 6:**
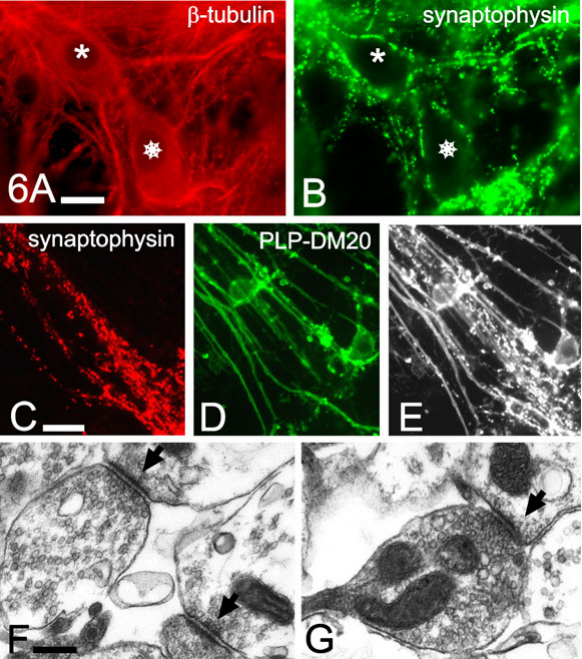
Abundant synapses form in the myelinating cultures. Cultures were co-stained at 30 days *in vitro* for neurons (A) or synaptophysin (B). The same neurons in A and B are indicated by an asterisk and a star. In C, D and E, the same region of the culture is immunostained for synaptophysin (C) and myelin sheaths (D) or co-labelled on the same image (E). Synaptophysin was abundantly expressed in a punctuate pattern around nerve cell bodies (A) and naked axons (C and E), but not in regions of myelin sheath formation (D and E). Cultures were also examined ultrastructurally for synapse formation (F and G); arrows indicate synapses. (A) β-Tubulin. (B and C) Synaptophysin. (D) Proteolipid protein (PLP)–DM20. (E) Synaptophysin and PLP-DM20. Scale bars: A and C, 10 μm; F, 250 nm.

Synaptophysin antigens were abundant around nerve cell bodies and along the length of neuronal processes, (Fig. 6A and B); however, synaptophysin staining was not observed in the myelinated regions of axons (Fig. 6C–E). Synapses were also readily identified by EM (Fig. 6F and G).

### Results of using other tissue sources for cultures

As the forebrain has a larger tissue volume, it was also trialled as a tissue source for myelinating cultures. Tissue from both E13.5 and E16.5 mouse fetuses was tried; the latter was selected, as development of the forebrain occurs at a later embryonic time point than that of the spinal cord. Cell survival of the dissociation procedure was good, but cells degenerated during the culture period, such that by 3 weeks, there were few surviving axons/neurons and no myelin, using either defined medium or co-culture medium (data not shown).

**Fig. 7:**
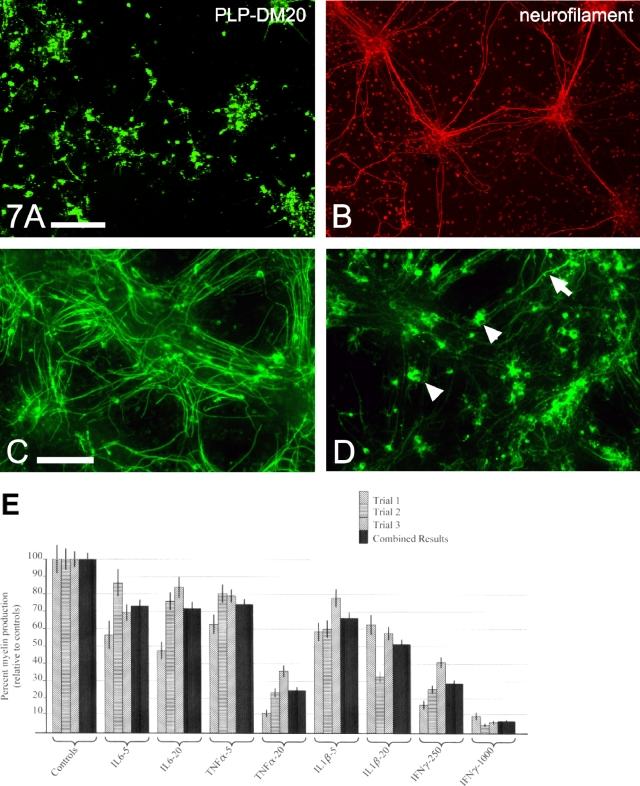
Illustration of results obtained from studies using rat embryonic tissue as a source for myelinating cultures (A and B) and the effect of certain cytokines on myelin formation in mouse cultures (C–E). (A and B) Cultures derived from rat embryo tissue at a cognate age of gestation to embryonic day 13.5 mouse embryos had poor cell survival, low axonal density and myelin sheath formation at 21 days *in vitro*. The same culture co-labelled for proteolipid protein (PLP)–DM20 (A) and phosphorylated neurofilament (B). Scale bar: 200 μm. To assess whether the cultures were a valid model for *in vitro* studies on the central nervous system, pro-inflammatory cytokines were added to the cultures and their effects on the formation of myelinated axons were determined at 24 days *in vitro*. (C) Control (non-treated) myelinating culture. (D) Culture treated with interferon-γ (IFN-γ) 1000 U/mL for 10 days during the period of active myelination. The arrow indicates a rare myelinated fibre developing in IFN-γ-treated culture; arrowheads indicate oligodendroglial cell bodies. Scale bar: 100 μm. (E) Graph depicting the effect of adding different cytokines to myelinating cultures. Results of individual trials and the combined results are shown. Units on the horizontal axis: interleukin-6 (IL-6), interleukin-1β (IL-1β) and tumour necrosis factor-α (TNF-α), ng/mL; IFN-γ, U/mL.

Rat embryonic spinal cord was also trialled as a source of tissue for myelinating cultures. Although the initial survival of cells in cultures derived from embryonic rat spinal cord was good, by 2 weeks *in vitro*, cell death was obvious, and by 3 weeks, survival of neurons and olidogendroglia was poor; consequently, formation of myelin was scarce (Fig. 7A and B).

### Effects of cytokines on myelination

To ascertain whether this method of generating myelinated cultures creates a valid model of the CNS for evaluating neuropathological conditions, the results of adding pro-inflammatory cytokines to myelinated axons were studied. Four different cytokines, each at two different concentrations, were added to the myelinating cultures, and the effects were determined by counting the number of myelinated axons crossing a superimposed grid. An example of the impact of a pro-inflammatory cytokine (IFN-γ) on myelination is compared with the non-treated controls in Fig. 7C and D. Cytokine experiments were performed in triplicate; thus, the total number of images in which myelinated axons were counted was between 160 and 180 for each experimental condition. The actual number of myelinated axons crossing the grid in the control (non-treated) cultures for the three experimental repeats was 18.2 ± 1.45 (average ± SEM), with a coefficient of variation of 7.97%. These data for the controls were consistent with the data derived in the baseline-13 dataset. The data comparing the effects of adding the cytokines to the cultures were expressed as percentage change of the control cultures in each experiment. The data from the three experimental repeats are presented in Fig. 7E. The combined results of the three experimental repeats show that the addition of each cytokine, at either dose, had an unequivocal, negative effect on myelination when compared to the control (non-treated) culture (*P* = 0.0001). Profound inhibition of myelination occurred with IL-1β 20 ng/mL, TNF-α 20 ng/mL, and IFN-γ at either dose (*P* ≈ 0). Three cytokines (TNF-α, IFN-γ and IL-1β) exhibited obvious dose–response curves; the effect of IL-6 is less clear, and requires further examination. The effects of the cytokines, at the different concentrations, were also compared to each other (Table 1). Some cytokines were found to have similar effects on myelination (e.g. TNF-α at 20 ng/mL and IFN-γ at 250 U/mL, not significantly different), whereas cytokines such as TNF-α at 20 ng/mL and IL-1β at 20 ng/mL had effects on myelination that were extremely significantly different (*P* < 0.0001) from each other (Fig. 7E, Table 1).

### Biochemical studies of myelin protein kinetics

A central objective of this study was to ascertain whether this system can generate material sufficient for detailed biochemical analysis of myelin protein production and the integration of such proteins into the myelin sheath. First, the total amount of protein recovered from the myelin fraction was determined. As myelin is a repository for key protein components, including MAG and PLP–DM20 (Griffiths *et al.*, 1998; Baumann & Pham-Dinh, 2001), the steady-state levels of these proteins were measured in the myelin fraction and total homogenate to assess their level of enrichment in the myelin fraction. The data were compared to data obtained from cultures of primary oligodendrocytes, in which cells develop extended myelin-like membranes in the absence of axonal contact (Benjamins & Nedelkoska, 1994).

**Fig. 8:**
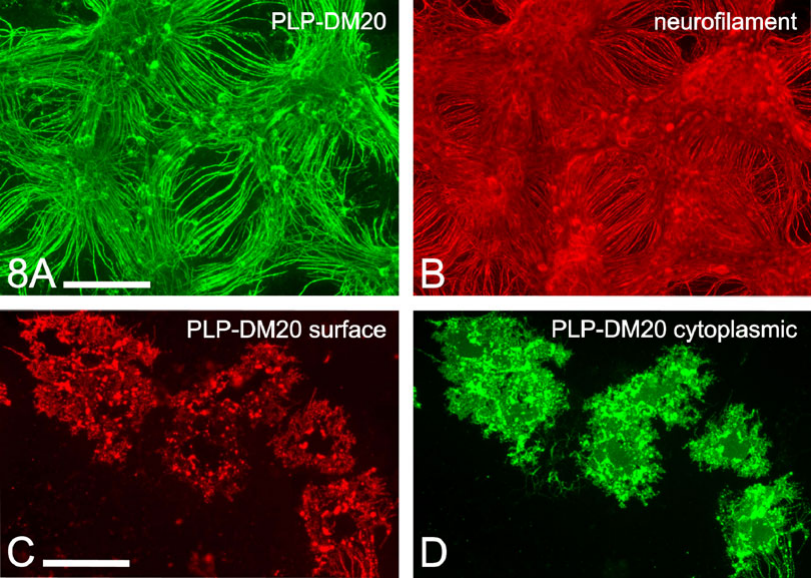
Examples of cultures used for biochemical preparations. Biochemical preparations were obtained from myelinating cultures derived from embryonic mice and grown for 33 days (A and B) and from dissociated neonatal mouse spinal cord oligodendrocytes, cultured for 7 days (C and D). Myelinating cultures were co-labelled for proteolipid protein (PLP)–DM20 (A) and phosphorylated neurofilament (B). Mouse oligodendroglia were co-stained for cell surface PLP–DM20 using the O10 antibody on live cells (C), and for cytosolic PLP–DM20 using AA3 antibody (D) after fixation and permeabilisation of cells. Scale bars: A and B, 200 μm; C, 50 μm.

Cell cultures (myelinating and oligodendrocyte) were established in Petri dishes and on coverslips to permit both biochemical and morphological analysis of each cell culture system. Myelinating cultures were harvested at 33 days *in vitro* for biochemistry and morphological evaluation (Fig. 8A and B). Dissociated oligodendrocytes were harvested at 7 days *in vitro*, by which time the oligodendrocytes had extended large myelin-like membranous sheets (Fig. 8C and D).

**Fig. 9:**
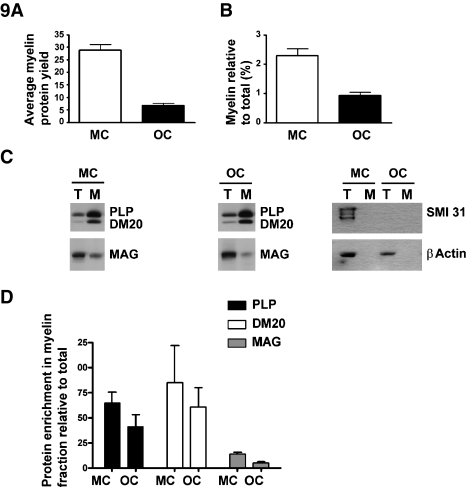

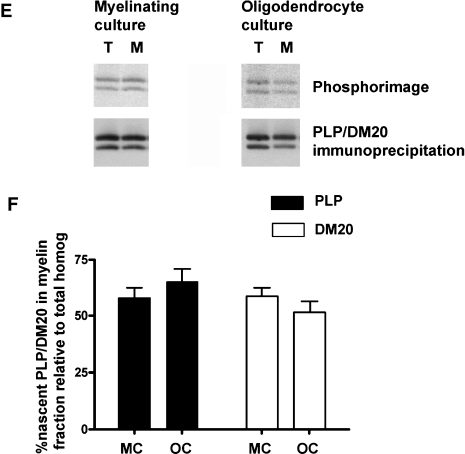
Myelin-enriched fraction can be isolated from myelinating cultures and used for myelin protein kinetic studies. (A) The data shown indicate the average protein yield (28.9 ± 2.2 μg) of the myelin fraction obtained from three experimental repeats for myelinating cultures (MC) as compared with the yield (6.7 ± 0.9 μg) from the myelin-like fraction isolated from oligodendrocyte cultures (OC). Each preparation was from one litter of five to seven embryos or five or six pups. Each tissue source yielded sufficient material to generate 6 × 35-mm^2^ Petri dish cultures. (B) The average protein recovered from the myelin fraction for each preparation is expressed relative to the total protein content of the homogenate. (C) Representative examples of western blot analysis of the two myelin proteins proteolipid protein (PLP)–DM20 and myelin-associated glycoprotein (MAG) detected in the total homogenate and myelin fraction from each culture type. The total homogenate (T) and the myelin fractions (M) were loaded onto the gel at a ratio of 20 : 1 (typically 20 μg and 1 μg respectively). Western blot staining for neuronal proteins (phosphorylated neurofilament) showed that there was no detectable contamination of the myelin fraction harvested from the myelinating cultures. It also showed that a significant proportion of the protein in the total cell homogenate of myelinating cultures is neuronally derived. The blots were stripped and reprobed with β-actin to assess equivalence of loading. (D) The intensity of the protein in the myelin fraction was corrected for the amount loaded onto the gel and expressed as the fold enrichment relative to the total homogenate intensity. (E) Nascent PLP and DM20 isoforms were incorporated into the myelin fraction. Representative phosphorimages of nascent PLP and DM20 immunoprecipitated from the myelin fraction and total homogenate are shown for each culture type. The nitrocellulose membranes were analysed by western blotting to compare the levels of PLP and DM20 isoforms recovered from each fraction. (F) The western blots were then quantitatively analysed by assessing the intensity of the signal for nascent PLP and DM20 isoforms relative to the total cellular homogenate. Note that the quantification is of the actual signal intensities in these blots, which were derived from loading 20 times more total cell homogenate then myelin fraction. Thus, if this 20-fold factor is taken into account, the absolute yield of PLP and DM20 in the myelin fraction is much higher than in the total cell homogenate.

A significantly higher protein yield was obtained from myelin isolated from the myelinating cultures (28.9 ± 2.2 μg per experiment) as compared to the oligodendrocyte culture system (6.7 ± 0.9 μg per experiment) (Fig. 9A). These protein yields were averaged from three separate preparations, using five to seven embryos for myelinating cultures and five or six pups for oligodendrocyte cultures. The protein yield obtained from the myelin fraction represents 2.3% ± 0.23% (myelinating cultures) and 0.93% ± 0.1% (oligodendrocyte cultures) of the total cellular protein that was pool harvested (Fig. 9B). This amount of material was sufficient for protein analysis by western blotting and also for isolation by immunoprecipitation. A comparison between the myelin yields relative to total cellular protein obtained between these two systems should be interpreted judiciously, as the total cellular protein from the myelinating cultures contains protein from other glia, neurons and axons, as indicated by the prominent staining for phosphorylated neurofilament in Fig. 9C. Conversely, the oligodendrocyte cultures are neuron-free and, at 7 days *in vitro*, have little contamination from other glia. Therefore, if the myelin yield could be compared to total cellular protein just derived from oligodendroglia, the yield would be proportionately much greater from the myelinating cultures than from the dissociated oligodendrocyte cultures. It is worth noting that we routinely obtain 0.38 ± 0.02 mg of protein from the myelin fraction isolated from the spinal cord of a normal P20 mouse. Such cords have a total protein content of approximately 11.4 ± 0.6 mg (where *n* = 3 ± SEM). The myelin protein yield from a P20 cord is calculated to be 3.3% of the total homogenate, which is in a similar range to the myelin protein yield obtained from the myelinating cultures.

Western blotting was used to determine the steady-state levels of PLP–DM20 and MAG in the myelin fractions obtained from both culture systems (Fig. 9C). As described in Materials and methods, a ratio of 20 : 1 total homogenate to myelin fraction had to be used to generate a signal intensity that was similar, and hence quantifiable, in each fraction. PLP and DM20 were found to be enriched in both cultures, with PLP being 64 ± 11-fold enriched in myelinating cultures and 40 ± 12-fold enriched in oligodendrocytes (Fig. 9D). MAG, on the other hand, was enriched 13.3 ± 2.2-fold in myelinating cultures and by a significantly lower factor of 5.0 ± 1-fold in myelin-like fraction from oligodendrocytes.

To determine whether this culture technique provides a valid model of the CNS *in vitro*, the neurobiology of myelin protein kinetics was studied. The incorporation efficiency of PLP–DM20 into the myelin fraction in both cell culture systems was investigated using ^35^S radiolabelling of nascent proteins. Fig. 9E depicts nascent PLP–DM20 that was immunoprecipitated from total cell homogenate or myelin extracts of both culture systems. Loading the gels at the 20 : 1 ratio described above generated robust signals from PLP and DM20 in both the total homogenate and myelin fraction. These signals derived from 20 : 1 loading ratios were quantified, and their intensity in the myelin fraction was expressed relative to the total fraction (Fig. 9F). There was no significant difference between the myelinating culture and the oligodendrocyte culture in the incorporation efficiency of either PLP (73% ± 16% and 65.1% ± 9.5%, respectively) or DM20 (72.7% ± 15% and 51.5% ± 8.8%). These values are similar to the incorporation efficiency of nascent PLP into the myelin fraction of brain slices of wild-type mice maintained in an artificial cerebrospinal fluid medium for up to 6 h (McLaughlin *et al.*, 2007).

## Discussion

For *in vitro* models of the CNS to be representative, they must accurately mimic the CNS *in vivo*, with respect to cell types and cellular interactions. Most biological or pathological studies on CNS myelin rely on *in vivo* experimentation, because *in vitro* methods have not reliably produced myelin in sufficient amounts for biochemical analysis. Our aim was to develop a method of culturing mammalian CNS tissue that is a close representation of the CNS *in vivo*. Our general specifications for the culture were for it to generate abundant myelinated axons and synapses, be technically easy, robust, and reproducible, have defined culture conditions, and facilitate visualisation of cellular events. Specifically, we wanted cultures that generated myelin in quantities sufficient for bulk biochemical analytical techniques. The method that we present here appears to fulfil all of these criteria. The morphological and biochemical data obtained from these cultures support the concept that this method recapitulates the CNS *in vitro*, and could be used to research biological and pathological mechanisms.

### Tissue source and culture media composition is critical

We examined the influences of embryonic age and media composition, with the aim of identifying the best myelinating conditions. For the cultures described in this article, E13.5 mice were the optimum tissue source. Tissue from younger embryos (E12.5) also yielded myelinating cultures, but a longer culture period was required, whereas tissue from E14.5 embryos had a greatly reduced cell yield. However, when rat embryos at a cognate stage to mouse development were used, survival of neurons was poor. In collaborative studies with A. Sorenson and S.C. Barnett (Glasgow), we have established a different method for producing myelinating rat spinal cord cultures, by culturing them on a monolayer of astrocytes (Sorensen *et al.*, 2008).

The composition of the medium is the other absolute determinant of culture success, as also shown in our previous study on spinal cord explants (Thomson *et al.*, 2006). The medium used here is relatively simple, has no growth factors *per se*, although it does have insulin, and has declining levels of serum for the first 12–13 days. Subsequently, the medium is insulin-free and completely defined (serum-free) during the critical period of myelin sheath formation, which begins around day 15 *in vitro*. This is the same medium that resulted in the best myelination and axonal growth in the explant cultures (Thomson *et al.*, 2006).

### Intercellular interactions are similar to those in in vivo systems

Myelination begins in the cultures at around 15–16 days, and is extensive by 22–28 days. Cultures can remain viable beyond 2 months. Myelin formation is widespread and of a comparable thickness to that obtained *in vivo*. The G-ratio, a measure of myelin sheath thickness relative to axonal diameter, of the myelin sheaths produced in our cultures was 0.76 ± 0.071 at 17–19 days after the onset of myelination. This value is comparable to measurements obtained from small-diameter axons in the optic nerve and large-diameter fibres from the ventral funiculus of the spinal cord of P20 mice (0.78 ± 0.06 and 0.711 ± 0.01, respectively) (I. R. Griffiths, J. E. Edgar, unpublished results).

Data established from 13 experimental repeats (number of images assessed 800) indicate consistent, reliable production of myelinated axons, across the whole of the culture area and between cultures.

Nodes of Ranvier are essential for rapid, saltatory conduction in the nervous system. As a consequence of becoming myelinated, the nodal, paranodal and juxtaparanodal domains of the axon are precisely organised at the molecular level to create the structural and functional properties of the nodes (Vabnick & Shrager, 1998). Nodes were identified frequently in the myelinating culture and had appropriate molecular specialisations of caspr and neurofascin. Caspr 1 (contactin-associated protein) is a major transmembrane component of the axo-glial junction at the paranode in both the peripheral nervous system and CNS, and may have a role in reciprocal signalling between the two cell types (Einheber *et al.*, 1997; Peles & Salzer, 2000). Its expression at the paranodes on either side of the node gives it the characteristic, doubled appearance indicative of well-formed perinodal domains. Neurofascins, expressed by neurons and glia, are also involved in axo-glial adhesion at the paranode (NF155) and in the structural specialisations of the adjacent node (NF186) (Tait *et al.*, 2000; Sherman *et al.*, 2005). The antibody that we used detects both neurofascins, so single domains of positive staining in the nodal/paranodal region were identified. The combined data consisting of the obvious interruptions in the myelin sheath (stained for PLP–DM20 or MBP) and the robust staining of the node/paranodal molecules caspr and neurofascin attest to an abundance of well-developed myelin sheaths in these myelinating cultures.

Synapse formation was extensive throughout the cultures, as indicated by synaptophysin expression and EM. Synaptophysin is a 38-kDa protein of the presynaptic vesicles of the CNS and neuromuscular junctions, and is a marker of synapse formation (Jahn *et al.*, 1985; Wiedenmann & Franke, 1985; Navone *et al.*, 1986; Calakos & Scheller, 1994; Berglund *et al.*, 2004). Synaptophysin expression occurred in these cultures where synapse formation would be expected, i.e. over neuronal cell bodies and processes, but not over myelin sheaths.

### Myelin can be extracted in sufficient quantities for biochemical investigations

The culture method described here produces myelin in sufficient quantities to permit its specific extraction and the subsequent analysis of key myelin components. We compared the data (total myelin recovery, myelin protein steady-state levels and enrichment of PLP–DM20 and MAG in the myelin fraction) from the myelinating cultures with that obtained from primary dissociated oligodendrocytes. This comparison provides an insight into the quality of the material extracted from the myelinating culture. There is a significantly greater yield of myelin fraction from the cultures in which true myelin sheath is formed than from the primary oligodendrocyte cultures in which just a myelin-like membrane is formed. This difference reflects the high density of compact myelin that forms in the myelinating culture as compared with the diffuse myelin-like, membranous sheets of the dissociated oligodendrocytes. The proportion of protein recovered in the myelin fraction relative to the total protein content from the myelinating culture (2.3%) was similar to that recovered from homogenised spinal cord tissue harvested from a P20 mouse (3.3%). Conversely, the proportion recovered from the myelin-like fraction of the oligodendrocyte cultures, in which all proteins are glial-derived (non-neuronal), was significantly less (0.93%). These data are consistent with the formation of adequate myelin, as indicated by the G-ratio data.

The myelin extraction protocol used in this study, and in many other laboratories, utilises differential centrifugal separation to isolate the lipid-rich myelin. Although the resulting myelin fraction is enriched in myelin lipids and proteins, it may also contain contaminants from the non-myelin fraction, including axolemmal components (Menon *et al.*, 2003). To confirm that the myelin fraction was enriched in myelin components, we analysed proteins associated with compact myelin (PLP–DM20), non-compact myelin (MAG) and axonal components. Both PLP and DM20, and to a lesser extent MAG, were greatly enhanced in the myelin fraction, and axonal contamination was not detected in this fraction. The greater abundance of PLP–DM20, as compared with MAG, in the myelin fraction is consistent with *in vivo* expression of these proteins in myelin. PLP–DM20 accounts for 50% of total myelin proteins in the CNS, whereas MAG comprises only 1% (Baumann & Pham-Dinh, 2001). A similar enrichment of PLP–DM20 was observed in the myelin-like fraction obtained from the oligodendrocytes.

### Validation of the cultures as in vitro models of the CNS for neurobiological and neuropathological research

Fundamental biological studies on myelin involve determining the kinetics of myelin protein production and incorporation of such proteins into the myelin sheath. The pathogenesis of dysmyelinating diseases can involve failure of transport and incorporation of key myelin proteins such as PLP–DM20 into myelin (McLaughlin *et al.*, 2006). The *rumpshaker* mouse has a dysmyelinating phenotype associated with *Plp1* mutation (Ile^186^Thr) (Schneider *et al.*, 1992) and serves as a model for human dysmyelinating disorders such as Pelizaeus–Merzbacher disease. Using an *in vitro* brain slice system, we have demonstrated previously that the incorporation of PLP–DM20 into myelin of *rumpshaker* mice is compromised as compared to wild-type mice (McLaughlin *et al.*, 2006). Kinetic studies on myelin proteins in brain slices use the myelin–axon units established in the living animal are hence valid representations of the *in vivo* situation. However, technical difficulties have limited such studies on myelin protein kinetics, as brain slices are only viable for a restricted time period, which we estimate to be up to 7 h. Using myelinating cultures prepared by the technique described in this article, we can now study myelin protein kinetics for unrestricted times, under defined conditions. A robust signal for radiolabelled nascent PLP–DM20 was detected in the myelin fractions isolated from both the myelinating and dissociated culture systems. The yield of labelled protein/culture was much greater from the myelinating cultures than from the oligodendrocyte cultures, permitting more extensive kinetic studies of myelin protein assembly and stability. Qualitative differences exist between compact myelin and myelin-like membranes, including differences in myelin proteins and lipids and the organisation of the membrane into lipid rafts (Marta *et al.*, 2003; Fitzner *et al.*, 2006). Consequently, the myelin-like membranes obtained from dissociated oligodendrocytes do not appear to be accurate biochemical representations of compact myelin. However, comparison of the data from the myelinating cultures with those from brain slices supports the hypothesis that the myelinating cultures generated by this technique are a valid representation of the CNS *in vitro* and can be used to study myelin protein kinetics. We are now using the myelinating cultures to dissect the mechanisms controlling myelin formation and maintenance in normal myelin and developmental disease conditions, such as in the dysmyelinating mouse mutant, *rumpshaker*.

An inflammatory response involving microglia, macrophage activation and T-cell invasion is a central feature of both developmental and acquired, myelin-related disorders such as Pelizaeus–Merzbacher disease and multiple sclerosis. The inflammatory response may precipitate myelin abnormalities (Ip *et al.*, 2006) or, conversely, it may promote remyelination (Setzu *et al.*, 2006). Teasing out the roles of individual events or specific molecules *in vivo* is challenging, due to individual animal variability. A major advantage of the technique described in this article is that the chemically defined conditions used during myelination enable precise examination of the impact of specific molecules, such as cytokines, on myelination. The method of assessment is robust and produces results that are highly statistically significant.

The roles of cytokines, such as IFN-γ, TNF-α, IL-1β and IL-6, expressed in inflammatory diseases such as multiple sclerosis are complex (Merrill & Benveniste, 1996; Sharief, 1998; Minagar & Alexander, 2003; Imitola *et al.*, 2005). They may be a direct, or indirect, cause of glial and myelin loss or axonal damage (Li *et al.*, 1993; Brosnan *et al.*, 1995; Parkinson *et al.*, 1997; McManus *et al.*, 1998; Sharief, 1998; Hesselgesser & Horuk, 1999; Huang *et al.*, 1999; Adorini, 2004; Imitola *et al.*, 2005; Zaheer *et al.*, 2007). We found that their addition to the cultures had highly significant, cytokine-specific and dose-dependent negative effects on myelin sheath formation, validating the use of this method of cell culture as a model for dissecting the molecular pathogenesis of CNS diseases. The data provided by these cytokine trials establish a model for investigating the effects of cytokines on myelin sheath formation as seen during developmental disorders, or during demyelinating diseases. Further studies would include adding lower doses of these cytokines, and/or combinations of cytokines, for a longer period during myelin formation. To investigate the role of cytokines in demyelinating diseases, cytokines could be added to the cultures after myelination has occurred (> 5 weeks *in vitro*.) Using this model, the specific effects of other molecules that are known to influence normal myelination, such as transferrin (Garcia *et al.*, 2004; Sow *et al.*, 2006), could be examined in more specific detail.

The technique described in this article for growing myelinating, synapsing CNS tissue *in vitro* is simple, defined, and robust. It permits easy morphological and biochemical analyses of cellular events associated with intercellular connections and myelination. This method of culturing CNS tissue should overcome many of the limitations of other CNS culture techniques, and advance research in areas of developmental neurobiology and CNS diseases, especially those involving myelin.

## Abbreviations

BSA: bovine serum albumin
CNS: central nervous system
DfM: differentiation medium
DMEM: Dulbecco’s modified Eagle’s medium
E: embryonic day
EM: electron microscopy
HBSS: Hank’s balanced salt solution
IFN-γ: interferon-γ
IL-1β: interleukin-1β
IL-6: interleukin-6
MAG: myelin-associated glycoprotein
MBP: myelin basic protein
P: postnatal day
PBS: phosphate-buffered saline
PLL: poly(l-lysine)
PLP: proteolipid protein
PM: plating medium
TNF-α: tumour necrosis factor-α
